# Items

**Published:** 1879-08

**Authors:** 


### Items.

THE AMERICAN ACADEMY OF MEDICINE.—This association of physicians was organized September, 1876, at Philadelphia, during the sessions of the International Medical Congress, when Traill Green, M.D., LL.D., of Easton, Pa., was elected its first President. Subsequently, meetings were held in New York (1877), and in Easton, Pa. (1878), at which Frank H. Hamilton, M.D., LL.D., of New York, and Lewis H. Steiner, A.M., M.D of Frederick, Md., were respectively chosen as Presidents. At these meetings the organization was more thoroughly perfected, and numerous accessions were made to the membership.

The objects of the Academy are thus broadly stated in its Constitution:

*First.* To bring those who are alumni of collegiate, scientific and medical schools into closer relations with each other.

*Second.* To encourage young men to pursue regular courses of study in classical or scientific institutions before entering upon the study of medicine.

*Third.* To extend the bounds of medical science, to elevate the profession, to relieve human suffering, and to prevent disease.

The following extract from the Annual Address of Dr. Frank H. Hamilton, President of the Academy, September, 1878, fully explains the purposes of its institution:

"The founders of this society sought, especially, by its organization, to aid others who are engaged in similar effort in this country, but who are working by other means, to remedy a great, and universally admitted evil, namely, imperfect preparation for the study of medicine, and its almost inevitable sequence, imperfect qualification on the part of those who are admitted to practice.

"There are many things which we can do more or less effectively. We can labor to create a sound public sentiment, which shall in some measure influence medical colleges and medical men, but more especially to create a sound sentiment among the young men who are contemplating the study and practice of medicine. They must be persuaded that it is unbecoming for them to enter upon the study of a learned profession without suitable classical and scientific knowledge, and without mental discipline; that it is impossible for them without this knowledge and discipline to make any respectable attainments in the science of medicine, and that it is shameful for them to enter upon the practice of medicine, and attempt to minister to the physical sufferings of their fellow-beings, without a competent knowledge of their science.

"Almost the entire medical profession in this country, including even most of that very large proportion who have not had the advantages of a thorough preliminary training, are urging its utility or necessity; the medical associations have, in all parts of the United States, again and again declared its importance, and especially is this true of the American Medical Association. The American medical journals have unanimously insisted upon radical changes in this respect; the professors and the alumni of medical colleges at their annual commencements, and in their social gatherings, have reiterated the same sentiment; but the work of reform in this direction is not yet accomplished. They need further help, and we have put our hands together to help them.

"Our association is not intended as a substitute for any other association of medical men; but we propose to supplement their labors. We fully believe that we can be useful in some small degree, and we shall not cease our efforts or disband our organization, until the needed reforms are accomplished."

The Fellows of the Academy must be Alumni of respectable collegiate institutions, who have received therefrom:

1. The degree of Bachelor of Arts, after a systematic course of study, preparatory and collegiate;

2. The degree of Master of Arts in accordance with the usage of these institutions;

3. The degree of Doctor of Medicine, after a regular course of study, not less than three years, under the direction and instruction of preceptors and professors. They must have also had an experience of three years in the practice of medicine.

Candidates for fellowship must be recommended by at least one Fellow, and be approved by a majority of the Council, after which the consent, by ballot, of two-thirds of the Fellows present will secure their election.

The initiation fee is $5.00, to be paid before initiation and registration.

Blank forms of application for fellowship can be obtained from the Secretary.

The annual meeting for 1879 will be held September 16, in New York. RICHARD J. DUNGLISON, M.D., *Secretary.*

P. O. Box 2386, Philadelphia.

HINDOO MEDICAL SCIENCE.— An appeal has been made by a Calcutta Baboo, named Rajendianath Datta, for a sum of 22,000 rupees wherewith to found a "free Ayur Veda College for the education of Hindoo physicians." The prospectus states "that in India, treatment, according to the Hindoo system of medicine, is generally more efficacious than that according to a foreign one. As our bodies are formed of and nourished by Indian materials, it would be against nature if medicines brought from foreign countries suit our constitution in time of illness. It is a fact greatly to be deplored that, owing to the influence of various social changes to which India has been subject, the hard earned gems of Hindoo medical science have become dim and soiled by disease, and are about to be lost and forgotten." If the Baboo cannot get the whole amount asked for, he is content to start on a smaller scale. That he relies stongly on the "gems of Hindoo science" is evidenced by the item in the monthly estimates for the purchase of "rare medical works from distant places," twenty-five rupees, which would certainly, at the present rate of exchange, represent a very modest library fund.—*Hosp. Gaz.*

